# Effect of Stretching on Ultraviolet Protection of Cotton and Cotton/Coolmax Blended Weft Knitted Fabric in a Wet State

**DOI:** 10.3390/ma7010058

**Published:** 2013-12-20

**Authors:** Chi-wai Kan, Lim-yung Yam, Sun-pui Ng

**Affiliations:** 1 Institute of Textiles and Clothing, The Hong Kong Polytechnic University, Hung Hom, Kowloon, Hong Kong 852, China; E-Mail: eddieyly@yahoo.com.hk; 2 Hong Kong Community College, The Hong Kong Polytechnic University, Hung Hom, Kowloon, Hong Kong 852, China; E-Mail: ccspng@hkcc-polyu.edu.hk

**Keywords:** cotton, Coolmax, weft knitted fabric, ultraviolet protection factor (UPF), wet, relax, stretching

## Abstract

In this paper, the ultraviolet protection factor (UPF) of plain knitted fabrics made from 20Ne cotton yarns, Coolmax yarn and their combinations in wet, relaxed and stretched states were studied. According to the fiber composition, fabric samples are divided into three groups including Group I (single cotton yarn), Group II (cotton/cotton combination) and Group III (Coolmax/cotton combination) for discussion. In order to study the effect of wet condition on the UPF of different plain knitted fabrics, five wetting solutions, namely: (i) chlorinated pool water; (ii) sea water, (iii) acidic perspiration; (iv) alkaline perspiration and (v) deionized water (DI water) were prepared and the fabrics were wetted with different percentages of 50%, 75% and 100%. The UPF of the plain knitted fabrics in wet, relaxed and stretched states was measured and the results were discussed. In addition, yarn and fabric properties such as yarn tenacity, yarn strength, fiber combination and water vapor transmission, which affect the corresponding UPF values, were used for generating a prediction model in order to determine UPF. Verification of the prediction model was also conducted.

## 1. Introduction

Jevtic (1990) found out that the T-shirt had a ultraviolet protection factor (UPF) of 15 and the surf shirt had a UPF of 36 but their UPF decreased by a factor of 1/3 when wetted [1]. When fabrics get wet, scattering is definitely reduced, leading to an increase of UV radiation penetration [1]. Osterwalder *et al.* [2] (2000) found out the average transmittance was about 30% for bleached cotton, while raised to 50% when wetted. Several researches supported that wetted fabric usually exhibited lower UPF values and variation of UV transmission because of the reduced optical scattering effect [1,3–6]. Several studies suggested that fabric under stretched conditions would generally decrease in UPF [7–9]. The rationale behind this is that the pores in the fabric structure are widened under stretch condition. However, reports on the UPF rating of knitted fabrics under stretched and wet conditions are seldom found. In daily activities, knitted fabrics will be subjected to different wet and stretch environment and their effects on UPF are of interest. As a result, the influence of stretching on UV protection of cotton and cotton/Coolmax blended weft knitted fabric in wet, relaxed and stretched states will be studied in this paper.

## 2. Experimental Section

### 2.1. Yarn Information

Grey cotton yarns and Coolmax yarn were supplied by The Central Textiles (H.K.) Ltd. (Hong Kong, China) and Shanghai Ming Mao Industrial Co., Ltd. (Shanghai, China) respectively. The yarn information is shown in Table 1. Three groups of yarn were prepared in order to study their effects on UPF: Group I (single cotton yarn), Group II (cotton/cotton combination) and Group III (Coolmax/cotton combination) and their combinations were shown in Table 2. In the case of cotton yarn, two types of yarn were used, conventional ring spun yarn and torque-free ring spun yarn. Torque-free ring spinning is a method of producing yarn with a torque reduction device in the conventional ring spinning system and the yarn structure is modified [10–13]. As indicated in Table 1, torque-free ring spun yarn had a lower yarn twist level than conventional ring spun yarn.

### 2.2. Weft Knitted Fabric Preparation

Based on the yarn combinations given in Table 2, 15 types of plain knitted fabrics were produced from DXC single jersey machine (Fukuhra, Japan). The knitting machine was 18 inches in diameter, with 54 feeders and 20 gauges with 2 cam tracks selection. Fabric samples were divided into three groups for study as mentioned in Table 2. The combined scouring and bleaching process was carried out as pretreatment and the treatment bath, containing Sandopan DTC (5 g/L), sodium hydroxide (10 g/L), Stabilizer AWN (1 mL/L) and 35% hydrogen peroxide (25 mL/L), was prepared. Fabric samples were padded with the liquor at 30 °C until 100% wet pick-up. Those padded fabric samples were steamed for 30 minutes at 105 °C and then they were rinsed thoroughly in hot and cold water. Finally, the fabric samples were laid flat and air-dried completely in standard conditioning environment (relative humidity of 65% ± 2% and temperature of 20 ± 2 °C) in order to avoid shrinkage during drying. After drying, the fabric samples were conditioned with relative humidity of 65% ± 2% and temperature of 20 ± 2 °C for at least 24 h before used.

### 2.3. Yarn Properties Measurement

All the yarn cones were conditioned with relative humidity of 65% ± 2% and temperature of 20 ± 2 °C for at least 24 hours before used. The yarn strength and tenacity was measured by USTER TENSORAPID 4 (Uster Technologies, Inc., Charlotte, NC, USA).

### 2.4. Water Vapor Transmission

Water vapor transmission (WVT) of knitted fabric samples was measured in accordance with ASTM E96 [15].

### 2.5. Preparation of Solutions to Simulate Wet Condition in Daily Use

Different wetting solutions were prepared to simulate different wet conditions in daily use: (i) chlorinated pool water (prepared according to AATCC 162 [16]); (ii) sea water (prepared according to EN ISO 105 E02) [17]; (iii) acidic perspiration (prepared according to EN ISO 105 E04) [18]; (iv) alkaline perspiration (prepared according to EN ISO 105 E04) [18] and (v) deionized water (DI water). After preparing the different solutions, the fabrics were treated with different pick-up of 50%, 75% and 100%, based on fabric weight, with the use of a padding machine.

### 2.6. Ultraviolet Protection Factor (UPF) Evaluation

#### 2.6.1. Evaluation under Wet and Relax Condition

UPF measurement was conducted by a Cary-300 Spectrophotometer (Varian Inc: Palo Alto, CA, USA). The AS/NZS 4399:1996 [19] was used and the UPF rating was derived from Equation (1). Readings of each fabric sample were taken from four positions and four times at each position (rotate 90° clockwise after each measurement). The average UPF values were then calculated from the readings.

$$\text{UPF}=\frac{\sum_{290}^{400}E_{\lambda }\times S_{\lambda }\times \Delta \lambda }{\sum_{290}^{400}E_{\lambda }\times S_{\lambda }\times T_{\lambda }\times \Delta \lambda }$$ (1)

where *E*_λ_ is relative erythemal spectral effectiveness; *S*_λ_ is solar spectral irradiance in Wm^−2^·nm^−1^; *T*_λ_ is spectral transmittance of the item; ∆λ is wavelength step in nm and λ is wavelength in nm.

#### 2.6.2. Evaluation under Wet and Stretch Condition

**Figure 1.**
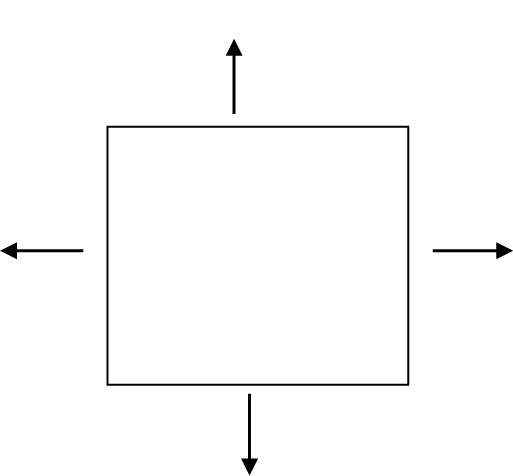
Way of stretching.

Stretching is another factor that may affect the UPF. Fabric samples were stretched in both lengthwise and cross-machine directions to 30% as shown in Figure 1.

### 2.7. Microscopy

Leica M125 stereomicroscope was used for the viewing the yarn with 12.5× magnification and for measuring the yarn diameter.

### 2.8. Prediction Model of UPF

SPSS was used to establish a prediction model by means of multiple linear regression (MLR) for UPF under different testing conditions. In this model, the dependent variable was UPF and the independent variables were yarn tenacity, yarn strength, fiber combination and water vapor transmission (WVT). A stepwise regression analysis with a confidence level of 95% was used in this study. Hence, those variables with p-values less than or equal to 0.05 were included in the final model whereas those with p-values greater than 0.05 were excluded. The significance of the prediction model was also evaluated by different tests in SPSS.

## 3. Results and Discussion

### 3.1. Group I (Single Cotton)—UPF at Wet and Relax State

**Figure 2.**
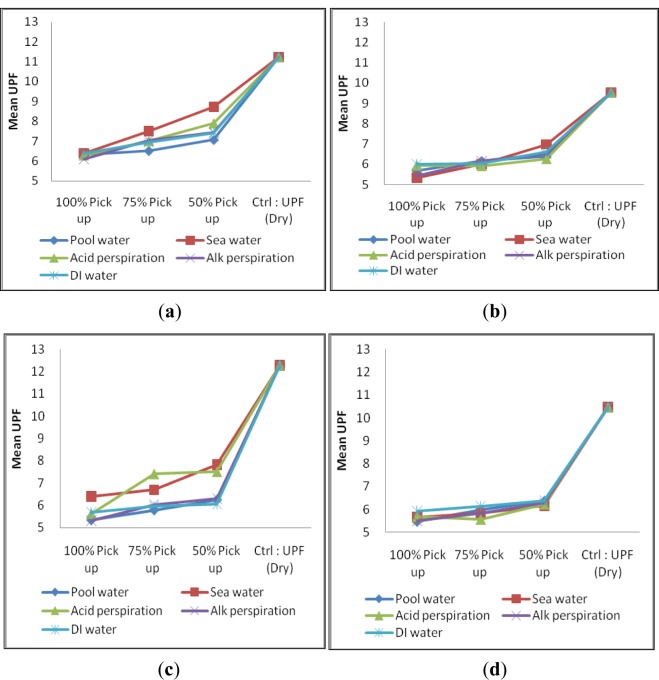
Ultraviolet protection factor (UPF) of samples in Group I: (**a**) CH; (**b**) MCG; (**c**) F and (**d**) MF wetted with five type of solution at three different pick-up percentages.

**Figure 3.**
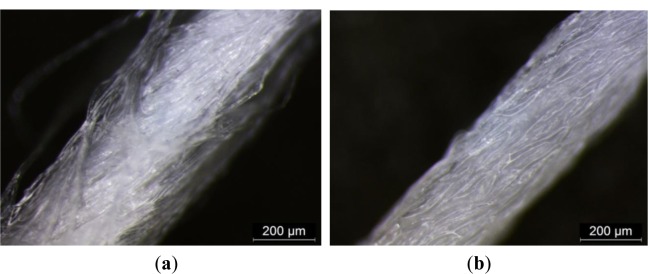

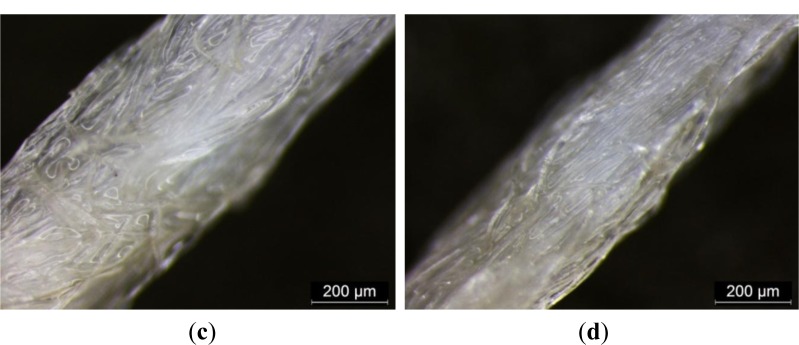
Microscopic view of (**a**) CH; (**b**) MCG; (**c**) F and (**d**) MF yarn wetted with D.I. water.

Samples CH, MCG, F and MF were wetted separately with five types of solution, *i.e.*, (i) chlorinated pool water; (ii) sea water; (iii) acidic perspiration; (iv) alkaline perspiration and (v) deionized water (D.I. water) with 50%, 75% and 100% pick-up based on the sample weight. With reference to Figure 2, it is observed that low pick-up percentage is associated with high UPF regardless of yarn type and it is applicable to the five solutions. Several researches supported that wetted fabric usually exhibits lower UPF values and variation of UV transmission because of the reduced optical scattering effect [3–6]. On the other hand, it is noted that a small variation of UPF was found on wetted torque-free ring spun yarn samples (Figure 2) when compared with conventional ring spun yarn samples (Figure 2). In addition, torque-free ring spun yarn samples generally provided lower UPF than conventional ring spun yarn after wetting. The small variation in UPF of torque-free spun yarns can be explained with reference to microscopic view of yarn types in Group I (wet state) as shown in Figure 3.

Yarn produced from the torque-free ring spinning method has in general less yarn twist numbers than conventional ring spun yarn [10–13]. The torque-free ring spun yarn is bulkier as it has less twist numbers to bind the fiber together when it is in a dry state. However, once the yarn is immersed in solution, the bulkiness presence in dry state disappears, as the surface tension of solution tends to pull fibers close together and eventually fill up the bulkiness. The yarn diameter of torque-free ring spun yarns (MCG and MF) becomes smaller and only swells at a smaller extend than conventional ring spun yarns (CH and F) when wetted. Changes in yarn diameter before and after wetting are shown in Table 3. UV radiation no longer passes through torque-free ring spun yarn as easily as it is in dry state on a single yarn level. The yarn diameter of torque-free ring spun yarn (Figure 3) becomes smaller in a wet state when compared to conventional ring spun yarn (Figure 3). Such observations help to explain smaller variations in UPF of torque-free ring spun yarn samples (MCG and MF) in wet state.

The reason for generally lower UPF of torque-free ring spun yarn sample (MCG and MF) could also refer to smaller yarn diameter after wetting. Fibers are closely bound with each other on a single yarn level. However, when the yarn diameter became smaller and holding all other factors being constant, the space and the hole in-between loops became bigger than before in dry state on the whole fabric level. This observation may help to explain why torque-free ring spun yarn made fabric sample could only yield comparative lower UPF than conventional ring spun yarn made fabric sample in wet state. However, no significant variation in UPF after picking up with different solutions of samples find within this group (Figure 2).

### 3.2. Group II (Cotton/Cotton Combination)—UPF at Wet and Relax State

**Figure 4.**
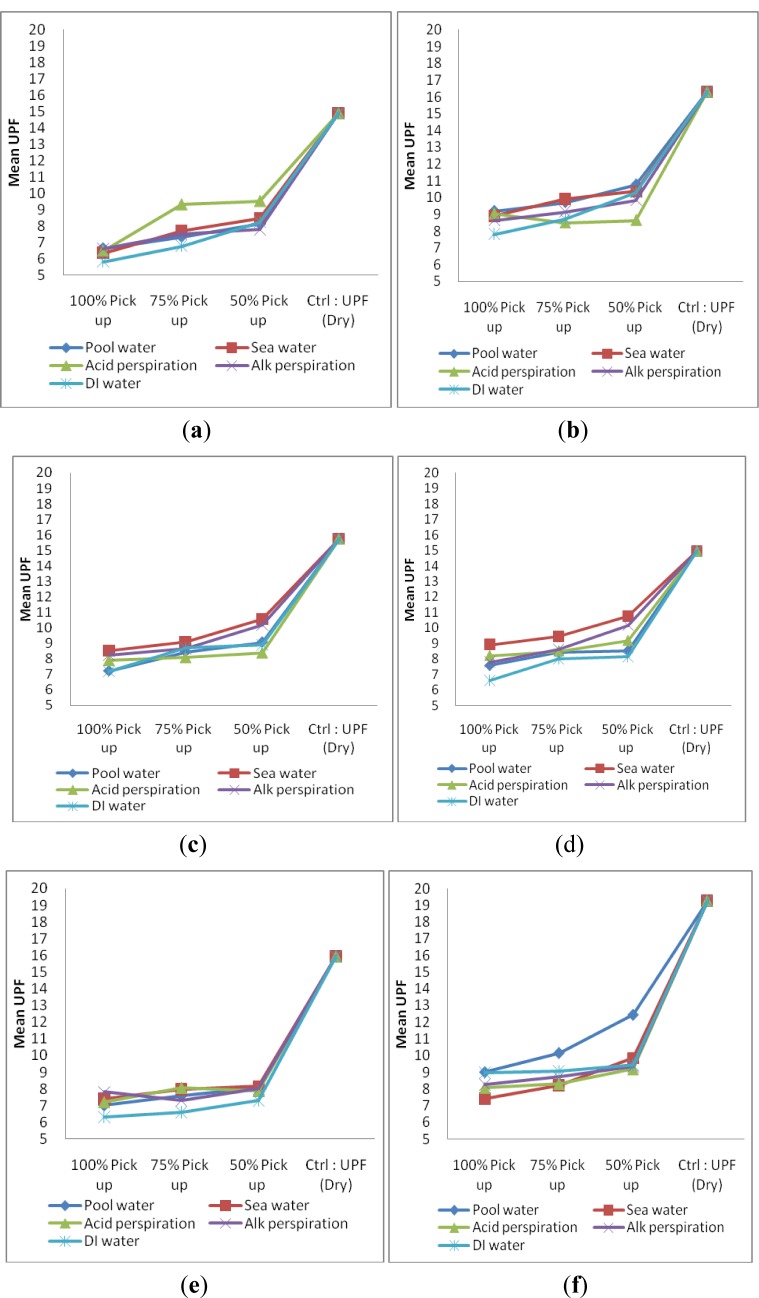
UPF of samples in Group II: (**a**) CH_MCG; (**b**) CH_F; (**c**) CH_MF; (**d**) MCG_F; (**e**) MCG_MF and (**f**) F_MF wetted with five types of solution at three different pick-up percentages.

There are six samples in Group II and their specifications are shown in Table 2. The overall performances regarding UPF in wet state of each sample of Group II are shown in Figure 4.

From Figure 4, it is noted that samples in Group II have a low pick-up percentage and can have a comparatively higher UPF value than high pick-up percentage, which is similar to the observation in Group I. Sample CH_MCG (combed cotton + combed cotton combination) is the one, which provides the lowest UPF rating in Group II. Another finding is only a small variation in UPF made from two torque-free ring spun yarns combination (MCG_MF, Figure 4e) when wetted with five types of solutions. It may be due to the reduction in yarn diameter together with relatively uniform fiber orientation (Figure 3) than wetted conventional ring spun yarn after wetting that may hinder UV radiation. No significant conclusion on the absorption of different solution among sample within Group II can be drawn as shown in Figure 4.

### 3.3. Group III (Coolmax/Cotton Combination)—UPF at Wet and Relax State

**Figure 5.**
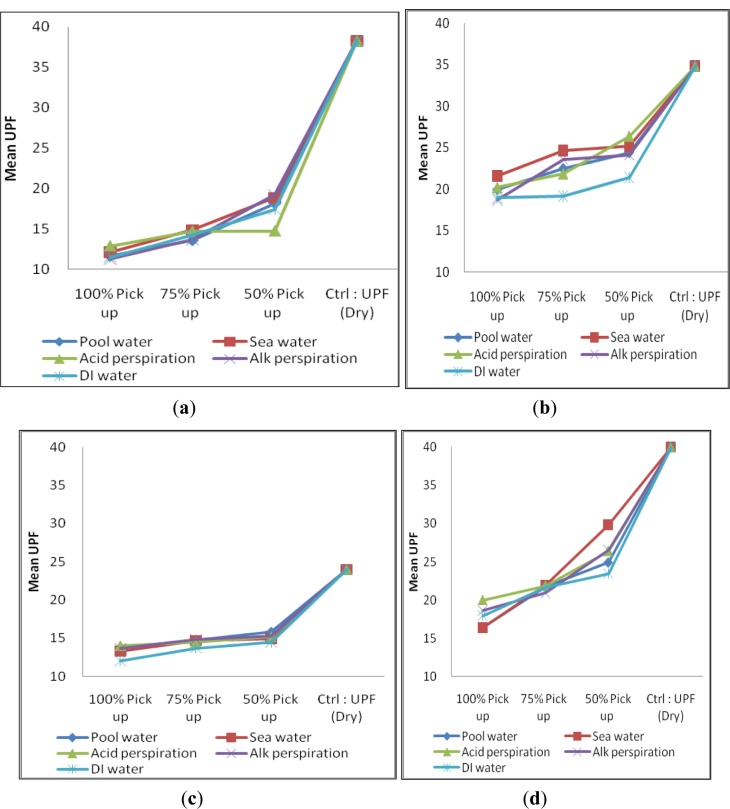

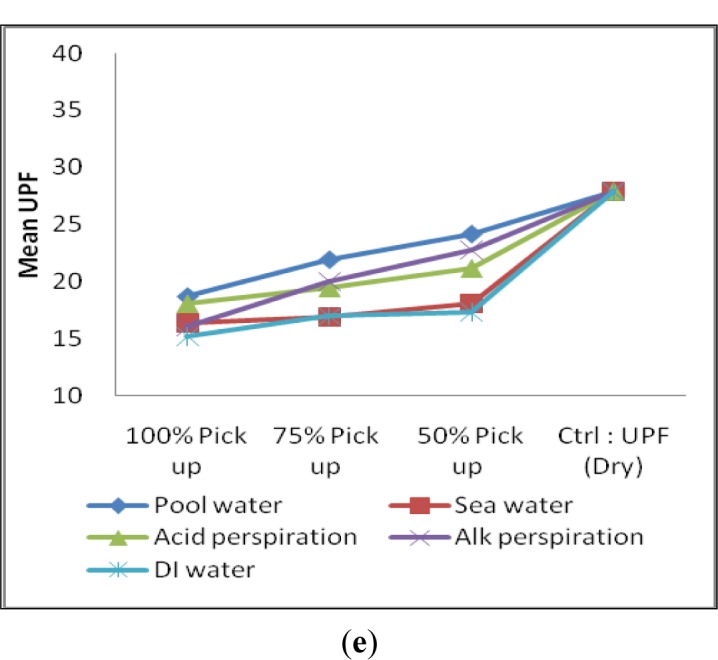
UPF of Group III samples: (**a**) CM; (**b**) CM_CH; (**c**) CM_MCG; (**d**) CM_F and (**e**) CM_MF wetted with five types of solution at three different pick-up percentages.

There are five samples in Group III and their specifications are shown in Table 2. Only sample CM is produced from pure Coolmax while the other four samples are Coolmax blended with different types of cotton yarns in Group I. The overall UPF results of fabric samples in Group III are shown in Figure 5.

The variation of UPF of sample CM (pure Coolmax sample) after wetting was comparatively low (Figure 5) when compared with the other Coolmax/cotton combinations. It can be explained by the pure Coolmax sample itself, which will not be affected by the influence of wetted cotton yarn, as it will not absorb solutions but only retain it. Coolmax is hydrophobic in nature and it is different from hydrophilic cotton, which will swell with the absorption of solution [20]. Similar to Group I and Group II fabric samples, fabric samples in Group III exhibit a relatively high UPF values when low pick-up percentage was used.

### 3.4. Prediction of UPF at Wet and Relax State (UPF_wet and relax_)

Yarn tenacity at break (tenacity), yarn strength, fiber combination and water vapor transmission (WVT) are used to compute and formulate Multiple Linear Regression (MLR) for predicting UPF at wet and relax state (UPF_wet and relax_). Equation (2) shows the proposed prediction model for UPF_wet and relax_.

$$\text{Y}=\text{a}+{\text{b}}_{1}(X_{1})+{\text{b}}_{2}(X_{2})+{\text{b}}_{3}(X_{3})+{\text{b}}_{4}(X_{4})$$ (2)

where Y is the UPF of wet and relax plain knitted fabric (UPF_wet and relax_); *X*_1_ is yarn tenacity (cN/tex); *X*_2_ is yarn strength (N); *X*_3_ is fiber combination (1: cellulose fiber, 2: cellulose combination, 3: synthetic fiber, 4: cellulose/synthetic combination) and *X*_4_ is water vapor transmission (WVT), b*_i_* (*i* = 1, 2, 3 and 4) is the related regression of *X_i_* (*i* = 1, 2, 3 and 4) and a is the intercept of the Equation (2).

UPF of samples wetted separately with 50%, 75% and 100% pick-up based on its weight and then averaged to derive an average value. It is difficult to determine the pick-up percentage on a particular part of the clothing during wearing, so the three pick-up percentages are averaged to get the average value, in order to become the dependent variable for prediction. UPFDI water was selected as the dependent variable for prediction, because clothing generally has a greater chance of coming in contact with water than the other solution types during daily use. By computing the relevant information using SPSS, the values of a, b_1_, b_2_, b_3_ and b_4_ can be found in the coefficient table as shown in Table 4. As shown in Table 4, yarn strength did not show statistical relationship with UPF_wet and relax_, as the *p*-value = 0.669 > 0.05. Therefore, yarn strength is excluded for the prediction model. As a result, the prediction model for UPF_wet and relax_ is shown in Equation (3) and the coefficient of multiple determination (*R*^2^) was found to be 0.859. This value means that 85.9% of the variation in the UPF_wet and relax_ can be explained by the variables of yarn tenacity, fiber combination and water vapor transmission.

$${\text{UPF}}_{\text{wet and relax}}=\text{14}.\text{575}+0.\text{283}X_{\text{1}}+\text{2}.\text{39}0X_{\text{3}}-\text{7}.\text{319}X_{\text{4}}$$ (3)

### 3.5. Verification of the Model Predictive Ability for UPF_wet and relax_

The UPF_wet and relax_ can be predicted by using yarn tenacity, fiber combination and water vapor transmission. In order to examine the precision of the model for prediction, verification of the model was conducted and the results are shown in Table 5.

Generally speaking, the prediction model tends to have good prediction of UPF_wet and relax_ and the average difference of all samples is −1.44%. The worst prediction is −22.72% on sample CH_F, while the best prediction is +0.41% on sample CM_MF. There are ten samples within 10% variation in the actual and predicted UPF values. The coefficient of determination (*R*^2^) of the model is 0.859, which means 85.9% of the total variances can be explained by the variables of yarn tenacity, fiber combination and water vapor transmission. The prediction model can be concluded as a successful way in predicting UPF_wet and relax_ state even for blended fiber combinations.

### 3.6. UPF at Wet and Stretch State

**Figure 6.**
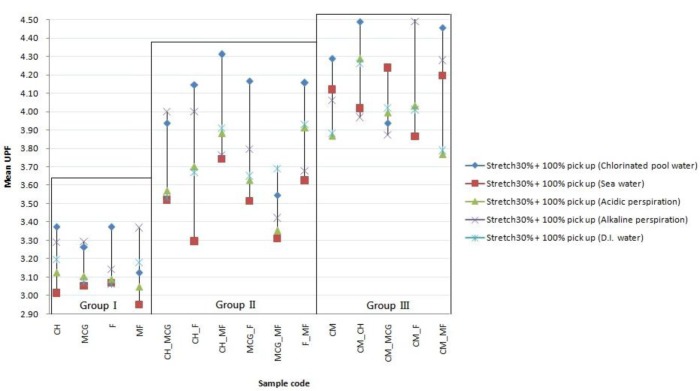
Overall performances of all samples subjected to 100% pick-up and 30% stretching.

According to previous research [2], the same fabric under wet and stretched condition would exhibit a remarkable decrease in UPF. After wetting the samples with solutions, the UPF dropped remarkable than in dry state. In order to understand a severe decrease in UPF when subjected to wetting and stretching at the same time, the most extreme condition was selected, *i.e*. (a) stretching 30% in both lengthwise and cross-machine directions and (b) wetting at 100% pick-up based on sample weight with the following solutions separately (i) chlorinated pool water; (ii) sea water; (iii) acidic perspiration; (iv) alkaline perspiration and (v) deionized water (DI water). The overall performance of three Groups is shown in Figure 6.

With reference to Figure 6, all samples are further reduced in their protective ability against UV radiation when subjected to wetting at 100% pick-up and 30% stretching in both lengthwise and cross machine directions at the same time.

The UPF_sea water_ is generally lower in Group I (single cotton) and Group II (cotton/cotton combination), no significant variation in Group III (Coolmax/cotton combination). It may be due to the surface rupture caused by sea water and thus reduce reflection. The observation further suggested sea water did not affect synthetic fiber as severe as cellulose fiber. The surface ruptures were increase of cotton fiber (cellulose fiber) after exposure to sea water, which is confirmed with the findings of Canetta *et al.* [21].

The UPF _chlorinated pool water_ is generally higher than the UPF after absorption of the remaining solutions, especially profound in Groups I and II, both of which are cotton fibers only. It may be explained by sodium hypochlorite is a kind of bleaching agent for whitening cotton fiber that may promote reflection.

Stretching may help to reveal the deteriorations brought out by solutions, as the UPF derive under both wetting and stretching show different results from different solution types.

### 3.7. Prediction of UPF at Wet and Stretch State (UPF _wet and stretch_)

It is difficult to determine the pick-up percentage on a particular part of the clothing during wearing, so the three pick-up percentages are averaged to get the average value to become the dependent variable for prediction model. Averaged UPF values from 50%, 75% and 100% pick-up of DI water and 30% stretching of sample was chosen as the dependent variable for prediction because clothing generally has greater chances come in contact with water and at the same time subjected to stretching during daily use. In this prediction, yarn tenacity, yarn strength, fiber combination and water vapor transmission are used to determine the prediction model of UPF at wet and stretch state (UPF_wet and stretch_) by Multiple Linear Regression (MLR). Equation (2) was used again for the proposed prediction model for UPF_wet and stretch_. By computing the relevant information using SPSS, the values of a, b_1_, b_2_, b_3_ and b_4_ can be found in the coefficient table as shown in Table 6. As shown in Table 6, yarn strength did not show statistical relationship with UPF_wet and stretch_, as the *p*-value = 0.699 > 0.05. Therefore, yarn strength is excluded for prediction. In addition, yarn tenacity is also excluded, as the *p*-value = 0.060 > 0.05, so yarn tenacity has no statistical relationship with UPF _wet and stretch_. As a result, only fiber combination and water vapor transmission will be used for the prediction model. The prediction model for UPF_wet and stretch_ is shown in Equation (4) and the coefficient of multiple determination (*R*^2^) was found to be 0.833. This value means that 83.3% of the variation in the UPF_wet and stretch_ can be explained by the variables of fiber combination and water vapor transmission.

$${\text{UPF}}_{\text{wet and relax}}=\text{1}.\text{922}+0.\text{394}X_{3}+0.\text{318}X_{4}$$ (4)

### 3.8. Verification of the Model Predictive Ability of UPF_wet and stretch_

The UPF_wet and stretch_ can be predicted by using fiber combination and water vapor transmission. In order to evaluate the precision of the prediction model, verification of the model was carried out and the results are shown in Table 7.

Generally speaking, the prediction model tends to have good estimation of UPF_wet and stretch_ and the overall differences of all samples are about −0.50%. The worst prediction is +7.47% on sample CH_F, while the best prediction is +0.03% on sample MCG. The actual and predicted UPF values of the samples are within 10% variation. The coefficient of determination (*R*^2^) of the model is 0.833 which means 83.3% of the total variance can be explained by fiber combination and water vapor transmission. The prediction model can be concluded as a successful way in predicting UPF_wet and stretch_ state even for blended fiber combinations.

## 4. Conclusions

Fifteen types of plain knitted fabrics were produced for this research and further divided them into three groups mainly based on the nature of fiber type. Group I consisted of single cotton fiber yarn, Group II consisted of two cotton fiber yarns combination while Group III consisted of cotton and Coolmax yarns combination.

In Group I, the effect of wetness could be concluded as being that the high pick-up percentage of solution (chlorinated pool water, sea water, acidic perspiration, alkaline perspiration and D.I. water) provided low UPF regardless of solution type, which could be further explained by the fact that wetness and retention of liquor reduced scattering. In addition, a lower UPF and a small variation of UPF were found on torque-free ring spun yarn in a wet state. The bulkiness of torque-free ring spun yarn presence in a dry state was bound by the surface tension of the solution when wetted and pulled fiber close together, and eventually filled up the bulkiness. Thus, UV radiation could no longer pass through torque-free spun yarn as easily as it is in dry state on a single yarn level. Comparative lower UPF of torque-free ring spun yarn could be explained by the yarn diameter becoming smaller in the samples MCG and MF (torque-free ring spun yarn sample) after wetting, in which the fibers are eventually closely pulled together on a single yarn level. When the yarn diameter became smaller and holding all other factors being constant, the space and the hole in-between loops were bigger than before, when it is in a dry state on the whole fabric level. This observation may help to explain the reason for torque-free ring spun yarn sample can only yield comparative lower UPF than conventional ring spun yarn sample in wet state.

In Group II, a low pick-up percentage provided a comparatively high UPF than a high pick-up percentage of each sample which was similar to Group I results. Of the two normal cotton combination samples, CH-MCG was the one that provided lowest UPF in Group II. Another finding was two torque-free ring spun yarn combinations: fabric sample MCG-MF behaved steadily when wetted, i.e. only a relatively small variation in UPF when wetted with five types of solutions. It may be due to a reduction in yarn diameter together with relatively uniform fiber orientation than wetted conventional ring spun yarn after wetting that may hinder UV radiation.

In Group III, the variation of UPF of sample CM (pure Coolmax sample) after wetting was comparatively small when compared with the other Coolmax/cotton combinations. It could be explained by the pure Coolmax sample itself, which will not affected by the influence of wetted cotton yarn as it will not absorb solutions but only retain them. Coolmax is hydrophobic in nature and it is different from hydrophilic cotton, which will swell with absorption of solution.

The UPF values further decrease when samples subjected to wet and stretched condition at the same time. Not only wetness on fiber generally would reduce scattering, but also pores were opened up when stretching thirty percentages in both machine and cross machine directions. It may explain the causes of further reduction in UPF.

## Figures and Tables

**Table 1.** Yarn specification [14].

Code	Fiber type	Spinning method	Twist number per 1 cm	Yarn count
CH	combed cotton	conventional ring spun	6.92	Ne 20
MCG	combed cotton	torque-free ring spun	4.68	Ne 20
F	combed supima cotton	conventional ring spun	5.38	Ne 20
MF	combed supima cotton	torque-free ring spun	4.20	Ne 20
CM	Coolmax	filament	1.03	150 dtex

**Table 2.** Yarn combinations [14].

Group	Code	Fiber type in yarn combination	Spinning method
Group I	CH	combed cotton	conventional ring spun
	MCG	combed cotton	torque-free ring spun
	F	combed supima cotton	conventional ring spun
	MF	combed supima cotton	torque-free ring spun

Group II	CH_MCG	combed cotton + combed cotton	conventional ring spun + torque-free ring spun
	CH_F	combed cotton + combed supima cotton	conventional ring spun + conventional ring spun
	CH_MF	combed cotton + combed supima cotton	ring spun + torque-free ring spun
	MCG_F	combed cotton + combed supima cotton	torque-free ring spun + conventional ring spun
	MCG_MF	combed cotton + combed supima cotton	torque-free ring spun + torque-free ring spun
	F_MF	combed supima cotton + combed supima cotton	conventional ring spun + torque-free ring spun

Group III	CM	Coolmax	filament
	CM_CH	Coolmax + combed cotton	filament + conventional ring spun
	CM_MCG	Coolmax + combed cotton	filament + torque-free ring spun
	CM_F	Coolmax + combed supima cotton	filament + conventional ring spun
	CM_MF	Coolmax + combed supima cotton	filament + torque-free ring spun

**Table 3.** Yarn diameter of yarns in Group 1 before and after wetting.

Code	Before wetting	After wetting	Increased by
CH	220 μm	410 μm	46.34%
MCG	225 μm	310 μm	27.42%
F	240 μm	440 μm	45.45%
MF	300 μm	380 μm	21.05%

**Table 4.** Coefficient table for model predicting UPF_wet and relax_.

Variable	Intercept/coefficient	Value	Significance	Interpretation
Constant	a	14.475	0.000	–
Yarn tenacity	b_1_	0.283	0.011	–
Yarn strength	b_2_	0.131	0.669	Exclude for prediction as *p* = 0.669 > 0.05, *i.e.* no significant linear relation with UPF_wet and relax_
Fiber combination	b_3_	2.390	0.000	–
Water vapor transmission	b_4_	−7.319	0.002	–

**Table 5.** Difference (%) between “Actual” and “Predicted” of UPF_wet and relax_.

Group	Sample code	UPF		Differences (%) between “Actual” and “Predicted”	
		Predicted	Actual		
Group I	CH	6.03	6.90	−	12.67%
	MCG	5.21	6.21	−	16.18%
	F	5.43	5.89	−	7.71%
	MF	6.36	6.14	+	3.55%
Group II	CH_MCG	7.86	6.93	+	13.30%
	CH_F	6.91	8.94	−	22.72%
	CH_MF	8.95	8.26	+	8.35%
	MCG-F	8.81	7.58	+	16.11%
	MCG_MF	7.07	6.73	+	5.07%
	F_MF	8.65	9.18	−	5.73%

Group III	CM	15.13	14.41	+	5.00%
	CM_CH	18.27	19.82	−	7.80%
	CM_MCG	14.31	13.36	+	7.08%
	CM_F	19.41	21.01	−	7.63%
	CM_MF	16.56	16.50	+	0.41%

**Table 6.** Coefficient table for model predicting UPF_wet and relax_.

Variable	Intercept/coefficient	Value	Significance	Interpretation
Constant	*a*	1.922	0.000	–
Yarn tenacity	*b*_1_	−7.319	0.060	exclude for prediction as*p* = 0.06 > 0.05, *i.e*., no significant linear relation with UPF_wet and stretch_
Yarn strength	*b*_2_	0.131	0.669	exclude for prediction as*p* = 0.669 > 0.05, *i.e.*, no significant linear relation with UPF _wet and stretch_
Fiber combination	*b*_3_	0.394	0.011	–
Water vapor transmission	*b*_4_	0.318	0.000	–

**Table 7.** Difference (%) between “Actual” and “Predicted” of UPF_wet and relax_.

Group	Sample code	UPF		Differences (%) between “Actual” and “Predicted”	
		Predicted	Actual		
Group I	CH	3.05	3.19	−	4.66%

	MCG	3.05	3.05	+	0.03%
	F	3.17	3.07	+	3.36%
	MF	3.07	2.95	+	4.18%

Group II	CH_MCG	3.45	3.52	−	2.04%
	CH_F	3.54	3.29	+	7.47%
	CH_MF	3.49	3.74	−	6.83%
	MCG_F	3.46	3.51	−	1.59%
	MCG_MF	3.51	3.69	−	5.01%
	F_MF	3.50	3.63	−	3.50%

Group III	CM	3.83	4.12	−	6.97%
	CM_CH	4.09	4.02	+	1.86%
	CM_MCG	4.26	4.24	+	0.40%
	CM_F	4.09	3.86	+	5.92%
	CM_MF	4.19	4.20	−	0.12%

## References

[1] Jevtic A.P. (1990). The sun protection effect of clothing, including beachwear. Aust. J. Dermatol.

[2] Osterwalder U., Schlenker W., Rohwer H., Martin E., Schuh S. (2000). Facts and fiction on UV protection by clothing. Radiat. Prot. Dosim.

[3] Gambichler T., Hatch K.L., Avermaete A., Altmeyer P., Hoffmann K. (2001). Sun protective clothes: Accuracy of laboratory testing. J. Eur. Acad. Dermatol. Venereol.

[4] Parisi A.V., Kimlin M.G., Mulheran L., Meldrum L.R., Randall C. (2000). Field-based measurements of personal erythermal ultraviolet exposure through a common summer garment. Photodermatol. Photoimmunol. Photomed.

[5] Moon R., Pailthorpe M. (1995). Effects of stretch and wetting on the UPF of elastance fabrics. Aust. Text.

[6] Paithorpe M. (1994). Textile and sun protection: The current situation. Aust. Text.

[7] Clark I.E.S., Grainger K.J.L., Agnew J.L., Driscoll C.M.H. (2000). Clothing protection measurements. Radiat. Prot. Dosim.

[8] Gies H.P., Roy C.R., Elliott G. (1992). Ultraviolet radiation protection factors for personal protection in both occupational and recreational situation. Radiat. Prot. Aust.

[9] Gies H.P., Roy C.R., Elliott G., Wang Z.L. (1994). Ultraviolet radiation protection factors for clothing. Health Phys.

[10] Kan C.W., Wong W.Y. (2011). Color properties of cellulase-treated cotton denim fabric manufactured by torque-free ring spun yarn. Text. Res. J.

[11] Murrells C.M., Tao X.M., Cheng K.P.S., Wong K.K. (2003). Production of torque-free singles ring yarns. Text. Asia.

[12] Tao X.M., Lo W.K., Lau Y.M. (1997). Torque-balanced singles knitting yarn spun by unconventional systems. Part I: Cotton rotor spun yarn. Text. Res. J.

[13] Tao X.M., Lo W.K., Lau Y.M. (1997). Torque-balanced singles knitting yarn spun by unconventional systems. Part II: Cotton friction spun DREF III yarn. Text. Res. J.

[14] Kan C.W., Yam L.Y., Ng S.P. (2013). The effect of stretching on ultraviolet protection of cotton and cotton/Coolmax blended weft knitted fabric in a dry state. Materials.

[15] American Society for Testing and Materials (2012). ASTM E96/E96M. Standard Test Methods for Water Vapor Transmission of Materials.

[16] AATCC Technical Manual (2011). AATCC Test Method 162, Colorfastness to Water: Chlorinated Pool.

[17] BSI Standards Publication (1996). EN ISO 105 E02. Colour Fastness to Sea Water.

[18] BSI Standards Publication (1996). EN ISO 105 E04. Colour Fastness to Perspiration.

[19] Australian/New Zealand Standard (1996). AS/NZS 4399: Sun Protective Clothing—Evaluation and Classification.

[20] Welo L.A., Ziifle H.M., Loeb L. (1952). Swelling capacities of fibers in water Part I: desiccation rate measurements. Text. Res. J.

[21] Canetta E., Montiel K., Adya A.K. (2009). Morphological changes in textile fibers exposed to environmental stresses: Atomic force microscopic examination. Forensic Sci. Int.

